# Association between variations in the *TLR4 *gene and incident type 2 diabetes is modified by the ratio of total cholesterol to HDL-cholesterol

**DOI:** 10.1186/1471-2350-9-9

**Published:** 2008-02-25

**Authors:** Melanie Kolz, Jens Baumert, Martina Müller, Natalie Khuseyinova, Norman Klopp, Barbara Thorand, Christine Meisinger, Christian Herder, Wolfgang Koenig, Thomas Illig

**Affiliations:** 1 Institute of Epidemiology, Helmholtz Center Munich, German Research Center for Environmental Health, Neuherberg, Germany; 2 Institute of Medical Information Processing, Biometry and Epidemiology, Ludwig-Maximilians-Universität, Munich, Germany; 3 Department of Internal Medicine II-Cardiology, University of Ulm Medical Center, Ulm, Germany; 4 Central Hospital of Augsburg, Germany; 5 Institute for Clinical Diabetes Research, German Diabetes Center, Leibniz Center at Heinrich Heine University, Düsseldorf, Germany

## Abstract

**Background:**

Toll-like receptor 4 (TLR4), the signaling receptor for lipopolysaccharides, is an important member of the innate immunity system. Since several studies have suggested that type 2 diabetes might be associated with changes in the innate immune response, we sought to investigate the association between genetic variants in the *TLR4 *gene and incident type 2 diabetes.

**Methods:**

A case-cohort study was conducted in initially healthy, middle-aged subjects from the MONICA/KORA Augsburg studies including 498 individuals with incident type 2 diabetes and 1,569 non-cases. Seven SNPs were systematically selected in the *TLR4 *gene and haplotypes were reconstructed.

**Results:**

The effect of *TLR4 *SNPs on incident type 2 diabetes was modified by the ratio of total cholesterol to high-density lipoprotein cholesterol (TC/HDL-C). In men, four out of seven *TLR4 *variants showed significant interaction with TC/HDL-C after correction for multiple testing (p < 0.01). The influence of the minor alleles of those variants on the incidence of type 2 diabetes was observed particularly for male patients with high values of TC/HDL-C. Consistent with these findings, haplotype-based analyses also revealed that the effect of two haplotypes on incident type 2 diabetes was modified by TC/HDL-C in men (p < 10^-3^). However, none of the investigated variants or haplotypes was associated with type 2 diabetes in main effect models without assessment of effect modifications.

**Conclusion:**

We conclude that minor alleles of several *TLR4 *variants, although not directly associated with type 2 diabetes might increase the risk for type 2 diabetes in subjects with high TC/HDL-C. Additionally, our results confirm previous studies reporting sex-related dissimilarities in the development of type 2 diabetes.

## Background

Subclinical, low-grade systemic inflammation has been implicated in the pathogenesis and prediction of type 2 diabetes. Several studies have shown that elevated levels of inflammatory and endothelial cell markers predict diabetes [1]. Both animal models and studies in humans have suggested that type 2 diabetes might be associated with changes in the innate immune response [1-3].

Toll-like receptors are members of the interleukin 1 receptor family, an evolutionary conserved signalling system against invading pathogens [4]. Due to their ability to recognize microbial components, mammalian toll-like receptors are among the most important components of the innate immunity pathway [5]. The first described and best-known member of this family is TLR4, identified as the signaling receptor for lipopolysaccharides [6]. TLR4 also interacts with endogenous ligands such as heat shock proteins [7], fibronectin, fibrinogen [8], minimally modified and oxidized low-density lipoprotein (LDL) [9,10] and free fatty acids [11], which are elevated in diabetes [12-14]. TLR4 ligation activates several intracellular signalling pathways, with TLR4/nuclear factor κB pathway being the most important one [15], leading to the synthesis and release of inflammatory cytokines and other costimulatory molecules that provide a link to adaptive immunity [16].

To date several single nucleotide polymorphisms (SNPs) have been identified in the *TLR4 *gene. Two of them, the congregated Asp299Gly and the Thr399Ile have been intensively studied and the rare allele 299Gly has been shown to cause hyporesponsiveness to lipopolysaccharides [17] and was reported to be associated with reduced incidence of carotid plaques and slowing progression of carotid atherosclerosis, as measured by carotid artery intima-media thickness [18].

Human genetic association studies of the *TLR4 *gene include only few that have assessed the relationship between genotype and type 2 diabetes and its complications and most of these studies have been restricted to those two polymorphisms, without consideration of the patterns of variation across the locus as a whole. Since a chronic low-grade inflammation seen in type 2 diabetes and in atherosclerosis may be related to common genetic predisposition and environmental risk factors, modulation of the systemic immune balance through gene polymorphisms might play a crucial role. Moreover, to our knowledge, there are no published prospective data evaluating the association between *TLR4 *variants and the development of type 2 diabetes. Therefore, we sought to systematically investigate the gender-specific association between genetic variants in the gene coding for the TLR4 receptor and incident type 2 diabetes in a prospective population-based case-cohort study. In addition, given that TLR4 has been reported to bind LDL [9,10], we wanted to analyze whether cholesterol levels have an effect on this association.

## Methods

### Study design

We designed a prospective case-cohort study within the population-based Monitoring of Trends and Determinants in Cardiovascular Disease (MONICA)/Cooperative Health Research in the Augsburg Region (KORA) Augsburg cohort study (1984–2002) [19]. As part of the international WHO MONICA project, three independent cross-sectional population-based studies (surveys) covering the city of Augsburg (Germany) and two adjacent counties were conducted in 1984/85 (S1), 1989/90 (S2) and 1994/95 (S3) to estimate the prevalence and distribution of cardiovascular risk factors among individuals aged 25 to 64 (S1) or 25 to 74 years (S2, S3). The study complies with the declaration of Helsinki. Approval was obtained by local ethic committees and informed consent was given from all patients. The total number of participants was 13,427 (6,725 men and 6,702 women). All subjects were prospectively followed within the framework of the MONICA/KORA studies [20]. The present study was restricted to subjects aged 35 to 74 years at baseline, since the incidence of type 2 diabetes is low in younger subjects. Altogether 10,718 persons (5,382 men and 5,336 women) of this age range participated in at least one of the three baseline surveys. After exclusion of 1,187 subjects with missing blood samples and 1,595 participants with prevalent type 2 diabetes, incident diabetes other than type 2 diabetes (e.g. type 1 or secondary diabetes), with self-reported, but not validated incident type 2 diabetes, without follow-up information or with a follow-up time of <1 year, the source population for the present study comprised 7,936 subjects (3,894 men and 4,042 women).

From the source population, a random sample was selected stratifying by sex and survey leading to a subcohort of 1,885 participants. After exclusion of subjects with missing DNA samples and missing values for risk factors, the final subcohort included 1,687 subjects (910 men, 777 women).

Additionally, all incident type 2 diabetes cases in the source population were selected, including subjects for whom the treating physician clearly reported the diagnosis or for whom the diagnosis was mentioned in the medical records or who were taking antidiabetic medication. The number of incident type 2 diabetes cases until December 31^st^, 2002 was 555 (329 men, 226 women). After exclusion of subjects with incomplete information on relevant variables, the present study including the subcohort and incident type 2 diabetes cases, was based on 2,067 participants (307 men, 191 women with incident type 2 diabetes; 835 men, 734 women without incident type 2 diabetes). Mean follow-up time (± SD) was 10.1 (± 4.9) years. The final stratum-specific sample sizes of this subcohort were used together with the stratum-specific sizes of the source population to compute sampling fractions, and the inverse of the sampling fractions yielded survey- and sex-specific sampling weights.

All cross-sectional analyses concerning SNP frequencies and tests for departures from Hardy-Weinberg-equilibrium were performed in a random sample of the whole study population with available DNA (i.e. without prior to exclusion of subjects without follow-up information, prevalent diabetes, etc.). This sample included 1,968 subjects (1,069 men, 899 women).

### Selection and genotyping of polymorphisms

For the SNP selection, the National Center for Biotechnology Information SNP database dbSNP Build 124 was used [21]. SNPs were chosen on the basis of density, frequency and occurrence in or near functional regions like exons and hypothetical promoter regions and hypothetical transcription factor-binding sites. In addition, all up to then known haplotype tagging SNPs were taken into account.

PCR primers were designed by Sequenom's MassArrayAssayDesign program. Genotyping analyses were carried out by means of matrix-assisted laser desorption ionization-time of flight analysis of allele dependent primer extension products as described elsewhere [22]. Genotyping calls were made in real time with MassArray RT software (Sequenom, San Diego, USA). Negative controls were included in all assays. To control for reproducibility of genotyping data, 12.5% of randomly selected samples were genotyped in duplicate. The discordance rate was 0.3%. Each SNP was tested for departures from Hardy-Weinberg-equilibrium by means of a chi-square test or Fisher's exact test depending on allele frequency.

### Assessment of demographic, lifestyle and clinical characteristics

Standardized interviews were conducted by trained medical staff (mainly nurses) to assess information concerning sociodemographic variables, smoking habits, leisure time physical activity level and alcohol consumption. In addition, participants underwent a standardized medical examination and a nonfasting venous blood sample was obtained. Detailed information on all survey methods has been described elsewhere in detail [23]. TC and HDL-C were measured by enzymatic methods (CHOD-PAP, Boehringer Mannheim, Germany). HDL-C was precipitated with phosphotungstic acid and magnesium ions.

### Statistical analysis

Means or proportions for baseline demographic and clinical characteristics were computed using the SAS procedures SURVEYREG or SURVEYFREQ which estimate standard errors appropriate to the sampling scheme. Tests of differences between subjects with and without incident type 2 diabetes were based on these procedures. In case of non-normality, tests were carried out with log-transformed variables and results were presented as geometric means with antilogs of standard errors of the log means.

Cox proportional hazards regression analysis was used to assess the association between polymorphisms within the *TLR4 *gene and incident type 2 diabetes. Due to the case-cohort design, standard errors were corrected using a "sampling weight" approach developed by Barlow (1994) [24]. Since sex-related differences seem to play a role in the development of diabetes [23,25], all analyses were done separately for men and women and carried out for each *TLR4 *SNP with a multivariate-adjusted model including age, body mass index (BMI), systolic blood pressure (SBP), TC/HDL-C, as well as the categorical variables survey, smoking status (never smoker, former smoker, current smoker), alcohol consumption (men 0, 0.1–39.9, ≥ 40 g/d; women 0, 0.1–19.9, ≥ 20 g/d) and physical activity (inactive vs. active, i.e. regular physical activity of ≥1 hour/week in both summer and winter). This model was respectively notated as "main effect model". To assess whether the impact of *TLR4 *variants on incident type 2 diabetes was modified by cholesterol levels, interaction terms of *TLR4 *variants and TC/HDL-C were additionally included to the main effect model ("interaction effect model"). Hazard ratios are presented with their 95% confidence intervals. P-values are based on robust variance estimates using the Barlow approach.

As measures for pairwise linkage disequilibrium (LD) between each pair of SNP loci, Lewontin's disequilibrium coefficient D' and the squared correlation coefficient were calculated. Haplotype reconstruction was performed within blocks of high D' using the expectation-maximization algorithm haplo.em [26]. To avoid large reconstruction errors resulting from missing data, haplotype estimation is based only on subjects with complete genotype information. Due to the study design, haplotype estimation for analysis of incident type 2 diabetes had to be performed separately for cases and non-cases. For association analysis within the population-based subcohort, no distinction had to be made for haplotype estimation. Haplotypes with frequencies <1% were collected into a separate group of rare haplotypes ("haplo rare"). The most frequent haplotype was used as the reference category. The effect of haplotypes on incident type 2 diabetes was assessed in an analogous way as for single SNPs. Due to the continuous coding of the expected number of haplotypes, an additive effect had to be assumed in haplotype association analysis.

The global significance level of 5% was corrected for the number of independent tests following the Bonferroni procedure. The number of independent tests was calculated as the number of effective loci obtained through spectral decomposition of the correlation matrix of all SNPs analyzed [27]. Therefore, the significance level for single tests was reduced to α = 0.01, corresponding to an overall significance level of α = 0.05. All statistical analyses were performed using the statistical package SAS Version 9.1 (SAS Institute, Cary, NC) and the statistical analysis software package R, Version 2.4.1 [28].

## Results

### Basic description of study population

The baseline demographic, clinical and lifestyle characteristics of the study participants are shown in Table 1. Subjects who developed type 2 diabetes during the follow-up period (cases) were older, showed a higher BMI, were more likely to be current or former smokers and were less active than subjects without onset of type 2 diabetes (non-cases). Furthermore, cases more frequently reported a history of myocardial infarction and hypertension, whereas no significant differences were observed for alcohol consumption between the two groups. As expected, TC and TC/HDL-C were considerably higher and HDL-C was considerably lower in cases compared to non-cases. Furthermore, cases had higher systolic and diastolic blood pressure compared to non-cases.

**Table 1:** Baseline demographic, lifestyle and clinical characteristics of the study participants during follow-up (n = 2,067). Data are weighted percentages for categorical variables and weighted means (standard errors) for normally distributed continuous variables.

	**Cases (N = 498)**	**Non-cases (N = 1569)**	*p*-value*
**Demographic**			
Sex = male [%]	61.7	53.2	<0.001
Age [yrs]	56.0 (0.4)	51.6 (0.3)	<0.001
**Clinical**			
Body Mass Index [kg/m^2^]	30.0 (0.2)	26.7 (0.1)	<0.001
Systolic Blood Pressure [mmHg]	142.2 (0.8)	132.7 (0.5)	<0.001
Diastolic Blood Pressure [mmHg]	85.3 (0.5)	81.5 (0.3)	<0.001
History of actual hypertension [%]	66.5	39.8	<0.001
History of myocardial infarction [%]	5.0	1.9	0.003
Total cholesterol [mg/dl]	247.4 (2.0)	235.9 (1.1)	<0.001
HDL cholesterol [mg/dl]	47.9 (0.6)	57.6 (0.4)	<0.001
Ratio TC/HDL-C	5.6 (0.1)	4.5 (0.0)	<0.001
**Lifestyle**			
Smoking status [%]			0.002
Current smoker	26.9	24.2	
Former smoker	33.3	27.2	
Never smoker	39.8	48.6	
Frequency of exercise [%]			<0.001
Inactive	69.1	58.3	
Active	30.9	41.7	
Alcohol consumption^† ^[%]			0.112
0 g/d	32.1	28.5	
0–39.9/0–19.9 g/d	39.4	44.6	
≥ 40/20 g/d	28.5	26.9	
**Survey**^‡^			<0.001
S1 [%]	36.5	28.9	
S2 [%]	39.4	36.1	
S3 [%]	24.1	35.0	

* t-test for continuous variables and chi-square-test for categorical variables; ^† ^Men: 0 g/d, 0–39.9 g/d, ≥40 g/d; Women: 0 g/d, 0–19.9 g/d, ≥20 g/d; ^‡ ^Independent cross-sectional study

### Association of TLR4 variants and haplotypes with type 2 diabetes in main effect models

Seven SNPs were genotyped in the *TLR4 *gene with a mean genotyping success rate of 98.7%. The assay for rs4986790 (Asp299Gly) failed, but it was tagged by rs4986791 (Thr399Ile). The characteristics of all SNPs are summarized in Table 2. The genotype frequencies of the analyzed SNPs were consistent with Hardy-Weinberg-equilibrium criteria. For three SNPs (rs4986791, rs7873784 and rs1927906) the homozygotes for the minor allele were pooled with the heterozygotes in the association analysis in order to avoid conclusions from low numbers, especially as results where analyzed stratified by gender. None of the SNPs in the *TLR4 *gene was significantly associated with incident type 2 diabetes in multivariate-adjusted models including age, BMI, SBP, TC/HDL-C, survey, smoking status, alcohol consumption and physical activity.

**Table 2:** Description of *TLR4 *variants genotyped (n = 1,968).

**SNP number**	**dbSNP identifier**	**Exchange**	**Genotype frequencies^a^**			**Gene location**
			
		**(1 > 2)**	**11**	**12**	**22**	
1	rs2770150	T > C	52.6	40.1	7.3	5'Upstream
2	rs6478317	A > G	46.5	42.7	10.8	5'Upstream
3	rs1927911	C > T	57.6	35.7	6.7	Intronic
4	rs2149356	C > A	47.7	42.2	10.1	Intronic, TFBS
5	rs4986791	C > T	88.1	11.6	0.3	Ile399Thr, HMCS
6	rs7873784	G > C	74.2	23.4	2.4	3'Untranslated region
7	rs1927906	A > G	81.3	18	0.8	3'Flanking

^a^weighted due to the sampling schene; TFBS: Transcription factor binding site; HMCS: human-mouse conserved segments

**Figure 1:**
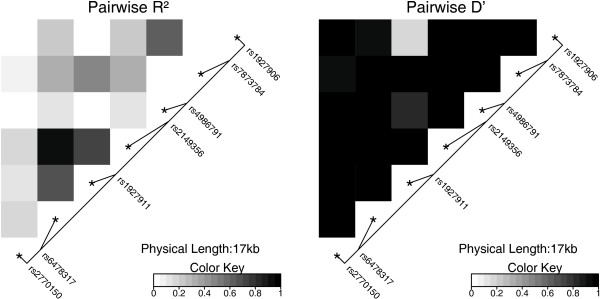
Structure of the *TLR4 *gene and pairwise LD *D' *und *r*^2 ^plots.

The LD structure across the *TLR4 *loci is shown in Figure 1. The strength of LD was reflected by restricted haplotype diversity. Three major haplotypes were observed with frequencies ranging from 13% to 41% (Table 3). The haplotype H1 carrying the major allele at all loci was used as the reference category. Similar to the analysis of the single SNPs, none of the haplotypes was associated with increased risk of incident type 2 diabetes.

**Table 3:** Description of *TLR4 *haplotypes in the randomly drawn subcohort (n = 1,968).

**Haplotype**	**SNP number**							**Frequency [%]**	
	**1**	**2**	**3**	**4**	**5**	**6**	**7**	**Men**	**Women**

H1	T	A	C	C	C	G	A	0.41	0.39
H2	C	A	C	C	C	G	A	0.27	0.28
H3	T	G	T	A	C	C	A	0.13	0.15
H4	T	G	T	A	C	G	A	0.07	0.07
H5	T	G	C	A	T	G	G	0.06	0.06
H6	T	G	T	A	C	G	G	0.04	0.03
H7	T	G	C	C	C	G	A	0.01	0.01
Rare								0.01	0.01

### Association of *TLR4 *variants and haplotypes with type 2 diabetes in interaction effect models

**Figure 2:**
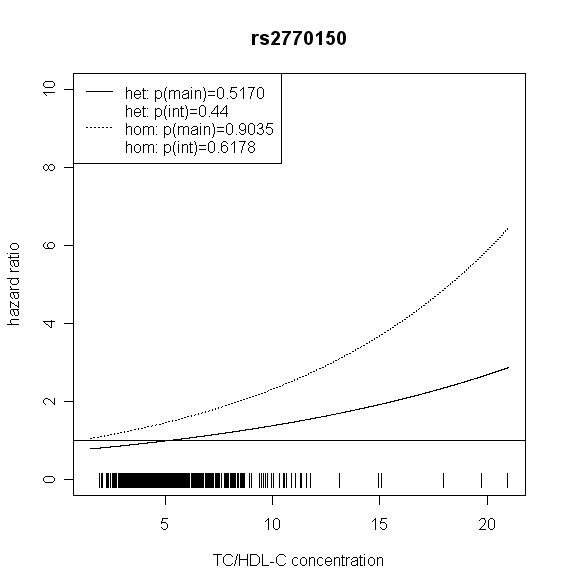
**Hazard ratios of incident type 2 diabetes related to the respective TC/HDL-C concentration in men for rs2770150**. Dashed lines represent subjects who are homozygote for the minor allele, while continuous lines show heterozygotes. Small bars at the x-axis indicate TC/HDL-C concentrations of patients with at least one copy of the minor allele.

**Figure 3:**
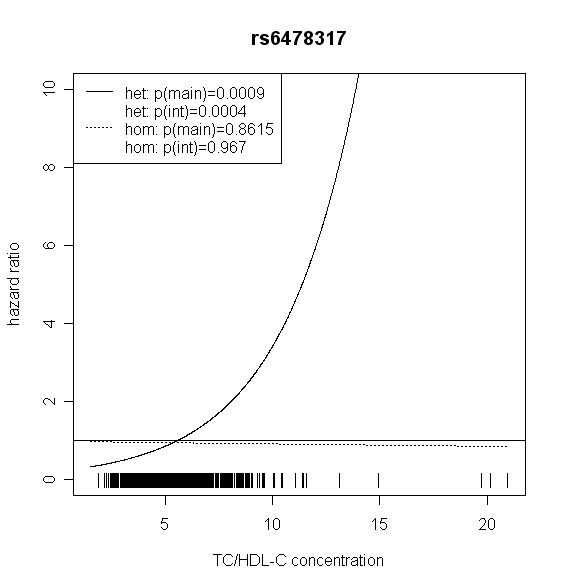
**Hazard ratios of incident type 2 diabetes related to the respective TC/HDL-C concentration in men for rs6478317**. Dashed lines represent subjects who are homozygote for the minor allele, while continuous lines show heterozygotes. Small bars at the x-axis indicate TC/HDL-C concentrations of patients with at least one copy of the minor allele.

**Figure 4:**
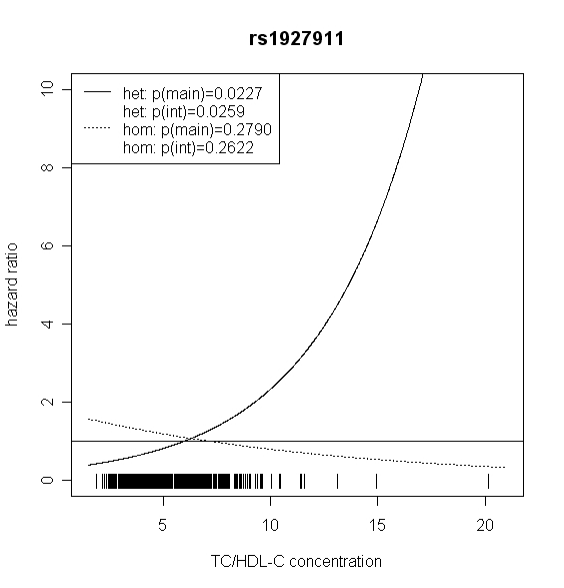
**Hazard ratios of incident type 2 diabetes related to the respective TC/HDL-C concentration in men for rs1927911**. Dashed lines represent subjects who are homozygote for the minor allele, while continuous lines show heterozygotes. Small bars at the x-axis indicate TC/HDL-C concentrations of patients with at least one copy of the minor allele.

**Figure 5:**
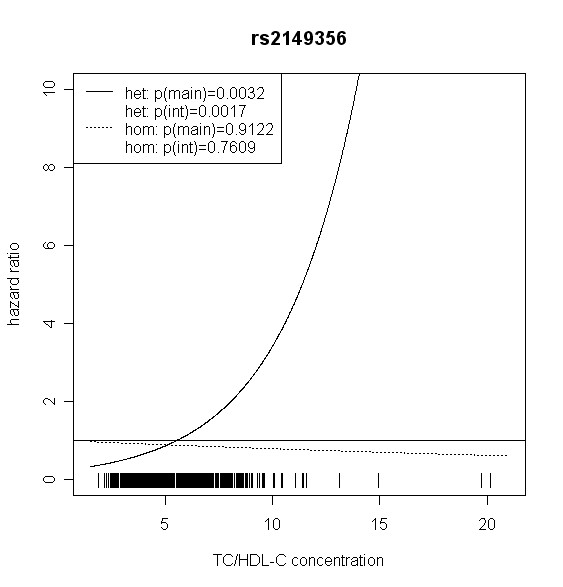
**Hazard ratios of incident type 2 diabetes related to the respective TC/HDL-C concentration in men for rs2149356**. Dashed lines represent subjects who are homozygote for the minor allele, while continuous lines show heterozygotes. Small bars at the x-axis indicate TC/HDL-C concentrations of patients with at least one copy of the minor allele.

**Figure 6:**
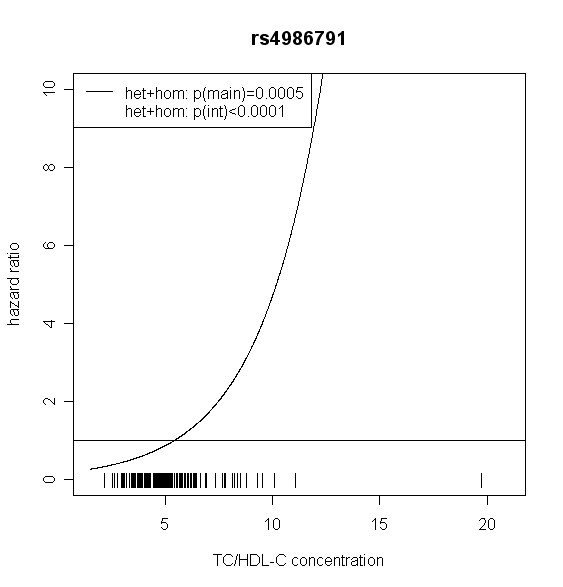
**Hazard ratios of incident type 2 diabetes related to the respective TC/HDL-C concentration in men for rs4986791**. Continuous lines represent subjects who are heterozygote or homozygote for the minor allele. Small bars at the x-axis indicate TC/HDL-C concentrations of patients with at least one copy of the minor allele.

**Figure 7:**
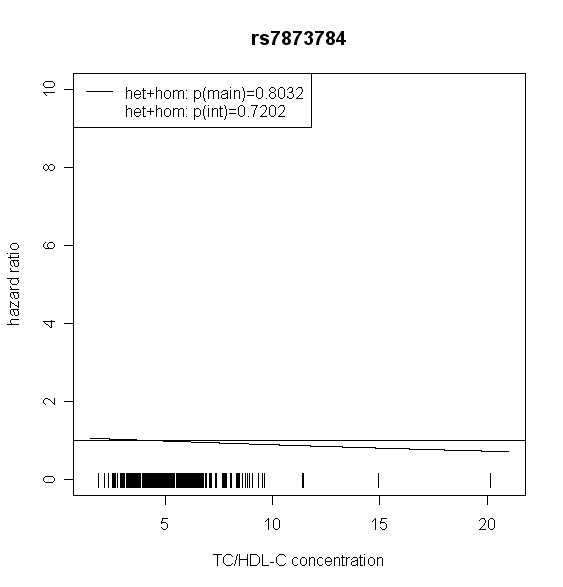
**Hazard ratios of incident type 2 diabetes related to the respective TC/HDL-C concentration in men for rs7873784**. Continuous lines represent subjects who are heterozygote or homozygote for the minor allele. Small bars at the x-axis indicate TC/HDL-C concentrations of patients with at least one copy of the minor allele.

**Figure 8:**
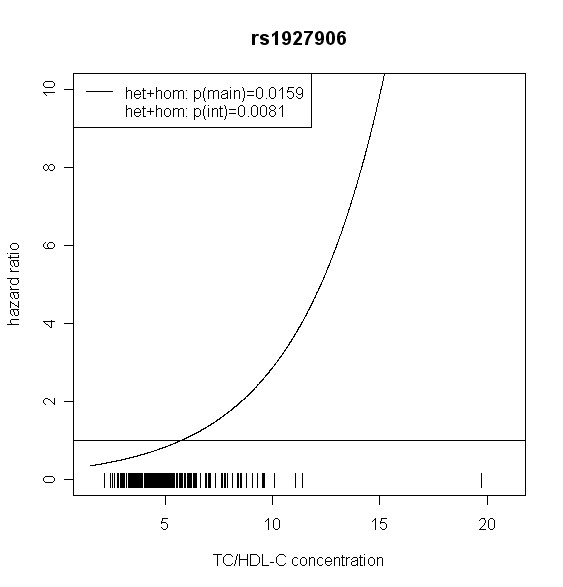
**Hazard ratios of incident type 2 diabetes related to the respective TC/HDL-C concentration in men for rs1927906**. Continuous lines represent subjects who are heterozygote or homozygote for the minor allele. Small bars at the x-axis indicate TC/HDL-C concentrations of patients with at least one copy of the minor allele.

**Figure 9:**
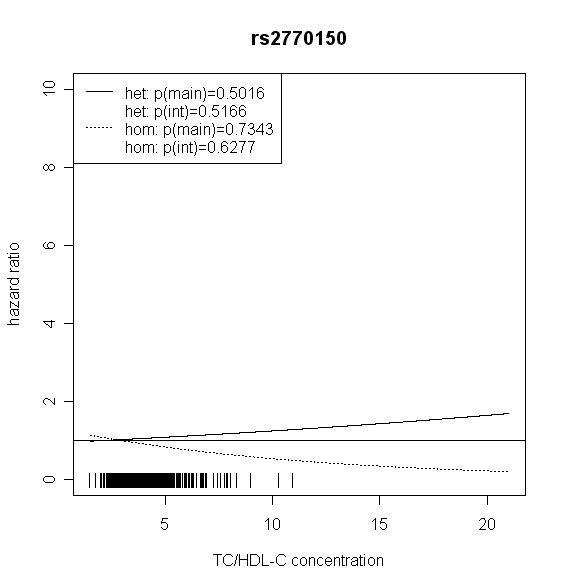
**Hazard ratios of incident type 2 diabetes related to the respective TC/HDL-C concentration in women for rs2770150**. Dashed lines represent subjects who are homozygote for the minor allele, while continuous lines show heterozygotes. Small bars at the x-axis indicate TC/HDL-C concentrations of patients with at least one copy of the minor allele.

**Figure 10:**
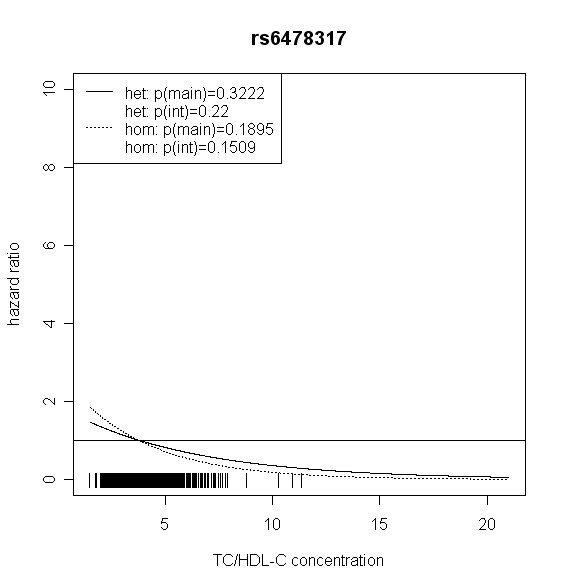
**Hazard ratios of incident type 2 diabetes related to the respective TC/HDL-C concentration in women for rs6478317**. Dashed lines represent subjects who are homozygote for the minor allele, while continuous lines show heterozygotes. Small bars at the x-axis indicate TC/HDL-C concentrations of patients with at least one copy of the minor allele.

**Figure 11:**
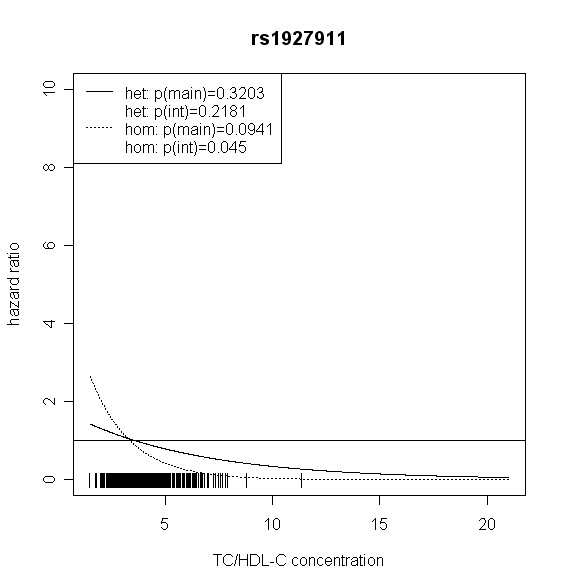
**Hazard ratios of incident type 2 diabetes related to the respective TC/HDL-C concentration in women for rs1927911**. Dashed lines represent subjects who are homozygote for the minor allele, while continuous lines show heterozygotes. Small bars at the x-axis indicate TC/HDL-C concentrations of patients with at least one copy of the minor allele.

**Figure 12:**
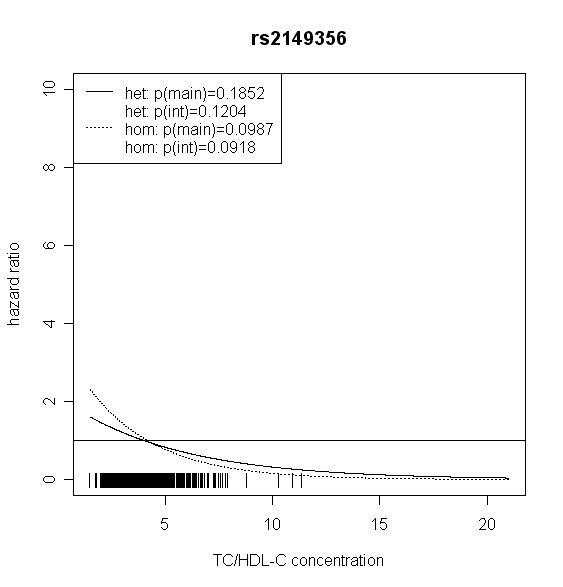
**Hazard ratios of incident type 2 diabetes related to the respective TC/HDL-C concentration in women for rs2149356**. Dashed lines represent subjects who are homozygote for the minor allele, while continuous lines show heterozygotes. Small bars at the x-axis indicate TC/HDL-C concentrations of patients with at least one copy of the minor allele.

**Figure 13:**
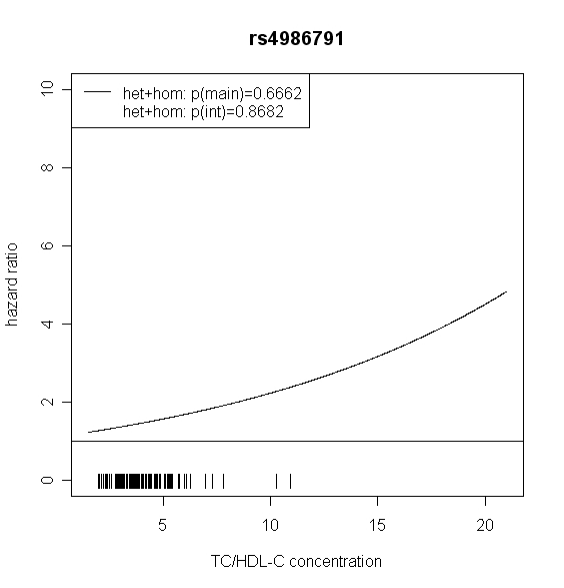
**Hazard ratios of incident type 2 diabetes related to the respective TC/HDL-C concentration in women for rs4986791**. Continuous lines represent subjects who are heterozygote or homozygote for the minor allele. Small bars at the x-axis indicate TC/HDL-C concentrations of patients with at least one copy of the minor allele.

**Figure 14:**
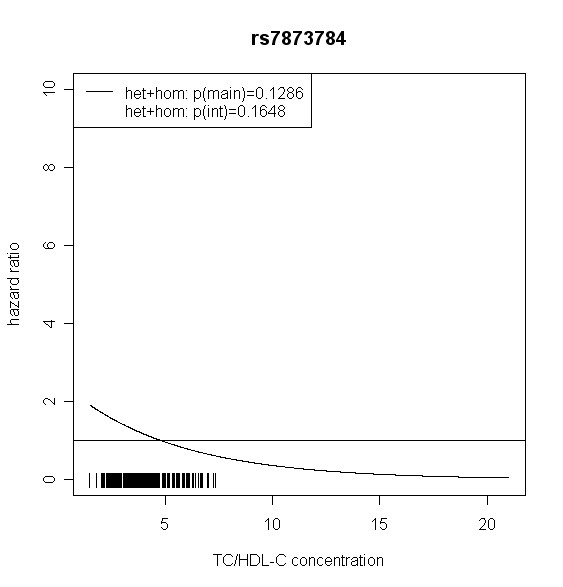
**Hazard ratios of incident type 2 diabetes related to the respective TC/HDL-C concentration in women for rs7873784**. Continuous lines represent subjects who are heterozygote or homozygote for the minor allele. Small bars at the x-axis indicate TC/HDL-C concentrations of patients with at least one copy of the minor allele.

**Figure 15:**
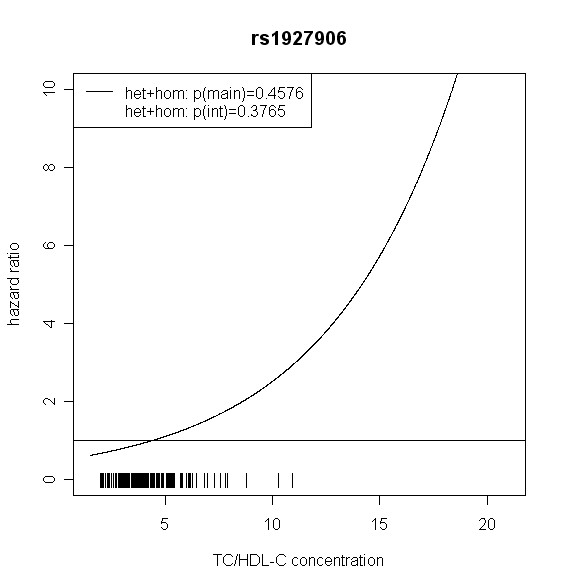
**Hazard ratios of incident type 2 diabetes related to the respective TC/HDL-C concentration in women for rs1927906**. Continuous lines represent subjects who are heterozygote or homozygote for the minor allele. Small bars at the x-axis indicate TC/HDL-C concentrations of patients with at least one copy of the minor allele.

The effect of *TLR4 *SNPs on incident type 2 diabetes was modified by TC/HDL-C in men. Five out of seven *TLR4 *variants showed significant interaction with TC/HDL-C with p values < 0.018 (test with two degrees of freedom). Four of them remained significant after correction for multiple testing (p < 0.01). In women, no significant effect modification by TC/HDL-C was observed. Hazard ratios of incident type 2 diabetes for each SNP related to the respective TC/HDL-C concentration are shown in Figures 2, 3, 4, 5, 6, 7, 8 for men and Figures 9, 10, 11, 12, 13, 14, 15 for women including the p-values for main and interaction effects above each figure.

For rs6478317 (Figure 3), rs1927911 (Figure 4) and rs2149356 (Figure 5), the hazard ratio of the heterozygote genotype compared to the homozygote genotype of the common allele increased with rising TC/HDL-C from below 1 (TC/HDL-C < 5) to 1.5 or more (TC/HDL-C > 8). Similar results were observed for rs4986791 (Figure 6) and rs1927906 (Figure 8) where heterozygotes and homozygotes for the minor allele were combined. We conducted several sensitivity analyses to ensure the observed associations. Further adjustment for lipid lowering drug intake or exclusion of participants with lipid lowering drug intake revealed very similar results. When analysing interactions excluding subjects with extreme TC/HDL-C values (below or above one percent of the distribution), the effect modifications remained but did not reach the level of statistical significance. The findings of the single SNP analysis were supported by the haplotype analysis shown in Table 4. In men, the effect of *TLR4 *haplotypes H5 and H7 on incident type 2 diabetes was modified by TC/HDL-C. Additionally, borderline significance was obtained for H4 in men and for H2, H3 and H4 in women. However, none of the *TLR4 *SNPs or haplotypes was associated with TC/HDL-C concentrations in the randomly drawn subcohort neither in men nor in women.

**Table 4:** Results of haplotype association analysis for type 2 diabetes in interaction effect models (n = 1,968).

	**Men**		**Women**	
	**HR [95% CI]**	**p-value**	**HR [95% CI]**	**p-value**

TC/HDL-C	1.02 [0.90; 1.15]	0.74	1.95 [1.55; 2.45]	1.2 × 10^-8^
H2	0.77 [0.36, 1.65]	0.5	3.11 [1.07; 9.00]	0.04
H3	1.04 [0.55; 1.96]	0.89	4.41 [1.37; 14.21]	0.01
H4	0.29 [0.08; 1.02]	0.05	4.01 [0.99; 16.17]	0.05
H5	0.11 [0.03; 0.45]	1.9 × 10^-3^	1.47 [0.34; 6.40]	0.61
H6	0.45 [0.11; 1.73]	0.24	0.29 [0.01; 6.73]	0.44
H7	0.05 [0.00; 0.52]	0.01	0.41 [0.02; 10.33]	0.59
TC/HDL-C × H2	1.08 [0.95; 1,24]	0.22	0.76 [0.61; 0.96]	0.02
TC/HDL-C × H3	1.01 [0.92; 1.10]	0.9	0.70 [0.54; 0.91]	0.01
TC/HDL-C × H4	1.28 [1.03; 1.58]	0.02	0.68 [0.51; 0.89]	0.01
TC/HDL-C × H5	1.51 [1.23; 1.87]	1.1 × 10^-4^	0.95 [0.70; 1.29]	0.74
TC/HDL-C × H6	1.14 [0.95; 1.37]	0.15	1.17 [0.65; 2.08]	0.61
TC/HDL-C × H7	1.63 [1.26; 2.12]	2.1 × 10^-4^	1.15 [0.64; 2.07]	0.64

In order to investigate the role of sex-specific differences and to verify that our findings are not artefacts resulting from stratified analysis, we also estimated models including three-way interaction terms between sex, TC/HDL-C and the genetic variants. These additional analyses gave evidence not only for presence of sex-specific differences of the influence of TC/HDL-C but also modification of genetic effects by sex, TC/HDL-C as well as sex-specific TC/HDL-C. For detailed results of the three-way interaction haplotype model, please see Additional file 1.

## Discussion

To the best of our knowledge, this is the first prospective study to systematically investigate the association between genetic variants in the gene coding for the TLR4 receptor and type 2 diabetes using a case-cohort design. In men the effects of *TLR4 *SNPs and haplotypes on incident type 2 diabetes were modified by TC/HDL-C concentrations. However, no effect was seen in women and none of the investigated SNPs or haplotypes was associated with type 2 diabetes alone.

### Association of *TLR4 *variants and haplotypes with type 2 diabetes in main effect models

There was no association between *TLR4 *SNPs or haplotypes and type 2 diabetes in our prospective population-based case-cohort study in main effect models.

Only few epidemiological studies have investigated the association of *TLR4 *polymorphisms with type 2 diabetes focusing only on the two classical polymorphisms Asp299Gly and Thr399Ile. We recently reported on the lack of an association between 299Gly and 3399Ile and prevalent type 2 diabetes or parameters of the metabolic syndrome using a cross-sectional approach [29]. A study in patients undergoing coronary angiography, found 299Gly carriage to be associated with a lower prevalence of diabetes [30]. However, this study represented a case-only study and did not distinguish between type 1 and type 2 diabetes. Additionally, carriers of the minor alleles of these two polymorphisms were associated with reduced prevalence of diabetic neuropathy in a sample of type 2 diabetes patients [31].

Evidence for a role of TLR4 in type 2 diabetes came from in-vitro studies. Levels of TLR4 have been reported to be consistently elevated in the diabetic NZL mouse model. Furthermore, dysregulated *TLR4 *mRNA expression correlates with high pro-inflammatory cytokine mRNA and low IL-10 levels after lipopolysaccharide stimulation [32]. During adipocyte differentiation, mRNA levels of TLR4 are remarkably enhanced in fat tissue of obese mice. Additionally, TLR4 activation provoked insulin resistance in adipocytes, suggesting that activation of TLR4 in adipocytes might be implicated in the onset of insulin resistance in obesity and type 2 diabetes [33].

### Association of TLR4 polymorphisms and haplotypes with incident type 2 diabetes modified by TC/HDL-C

In the present study, the effect of *TLR4 *SNPs on incident type 2 diabetes was modified by TC/HDL-C in men, but not in women. To our knowledge, this effect has not been described before. An influence of the minor alleles of four TLR4 variants on the incidence of type 2 diabetes was observed particularly for patients with high levels of TC/HDL-C and these findings were supported by haplotype analysis. In men, two haplotypes H5 and H7 showed a significant interaction with TC/HDL-C on the risk of incident type 2 diabetes. H5, the haplotype showing the strongest effect is tagged by the rare allele of the common Thr399Ile polymorphism.

The effect of Asp299Gly and Thr399Ile on coronary artery disease was evaluated in a randomized cholesterol-lowering trial. The authors reported a significant interaction between genotype and statin treatment in reducing the risk of clinical cardiovascular events. Furthermore, like in our sample, they did not observe an effect on lipid parameters alone [34].

Several in-vitro and animal studies have focused on the possible activation of the TLR pathways by hyperlipidemia. Oxidized LDL induces upregulation of TLR4 expression in macrophages in vitro and might therefore contribute to the TLR4-dependent inflammatory process in the arterial wall [10]. An in-vitro study revealed a mechanism by which activation of TLR4 results in a strong inhibition of cholesterol efflux from macrophages [35]. Additionally, mouse models of hyperlipidemia have suggested a role for the TLR signaling pathways in hyperlipidemia-induced atherosclerosis [36].

### Limitations and strengths of the present study

We studied a large cohort of middle-aged men and women of German nationality. Therefore replication in other populations is needed before results can be generalized. Strengths of the study include a population-based prospective study design with a long follow-up and the detailed systematic selection of polymorphisms using LD information available in public databases and the reconstruction of haplotypes.

## Conclusion

We conclude that minor alleles of several *TLR4 *variants, although not directly associated with type 2 diabetes might increase the risk for type 2 diabetes in subjects with high TC/HDL-C. Our results confirm previous studies reporting sex-related dissimilarities in the development of type 2 diabetes. However, further studies are needed to replicate these results and analyse the underlying mechanisms.

## Competing interests

The author(s) declare that they have no competing interests.

## Authors' contributions

MK participated in the in the molecular genetic studies and drafted the manuscript. JB performed the statistical analysis. MM participated in the statistical analysis and performed haplotype reconstruction. NK participated in the design of the study. NK carried out the molecular genetic studies. BT participated in the design of the study and helped to draft the manuscript. CM participated in the design of the study and helped to draft the manuscript. CH participated in the design of the study. WK conceived of the study, and participated in its design and coordination. TI conceived of the study, and participated in its design and coordination. All authors read and approved the final manuscript.

## Pre-publication history

The pre-publication history for this paper can be accessed here:



## Supplementary Material

Additional file 1 Results of interaction effect models including three-way interaction terms between sex, TC/HDL-C and the genetic variants. Results of haplotype and SNP association analysis for men and women combined including three-way interaction terms between sex, TC/HDL-C and the genetic variants. Click here for file

## References

[B1] Kolb H, Mandrup-Poulsen T (2005). An immune origin of type 2 diabetes?. Diabetologia.

[B2] Duncan BB, Schmidt MI (2006). The epidemiology of low-grade chronic systemic inflammation and type 2 diabetes. Diabetes Technol Ther.

[B3] Pickup JC, Crook MA (1998). Is type II diabetes mellitus a disease of the innate immune system?. Diabetologia.

[B4] Medzhitov R, Preston-Hurlburt P, Janeway CA (1997). A human homologue of the Drosophila Toll protein signals activation of adaptive immunity. Nature.

[B5] Barton GM, Medzhitov R (2003). Toll-like receptor signaling pathways. Science.

[B6] Poltorak A, He X, Smirnova I, Liu MY, Van Huffel C, Du X, Birdwell D, Alejos E, Silva M, Galanos C, Freudenberg M, Ricciardi-Castagnoli P, Layton B, Beutler B (1998). Defective LPS signaling in C3H/HeJ and C57BL/10ScCr mice: mutations in Tlr4 gene. Science.

[B7] Ohashi K, Burkart V, Flohe S, Kolb H (2000). Cutting edge: heat shock protein 60 is a putative endogenous ligand of the toll-like receptor-4 complex. J Immunol.

[B8] Smiley ST, King JA, Hancock WW (2001). Fibrinogen stimulates macrophage chemokine secretion through toll-like receptor 4. J Immunol.

[B9] Miller YI, Viriyakosol S, Binder CJ, Feramisco JR, Kirkland TN, Witztum JL (2003). Minimally modified LDL binds to CD14, induces macrophage spreading via TLR4/MD-2, and inhibits phagocytosis of apoptotic cells. J Biol Chem.

[B10] Xu XH, Shah PK, Faure E, Equils O, Thomas L, Fishbein MC, Luthringer D, Xu XP, Rajavashisth TB, Yano J, Kaul S, Arditi M (2001). Toll-like receptor-4 is expressed by macrophages in murine and human lipid-rich atherosclerotic plaques and upregulated by oxidized LDL. Circulation.

[B11] Shi H, Kokoeva MV, Inouye K, Tzameli I, Yin H, Flier JS (2006). TLR4 links innate immunity and fatty acid-induced insulin resistance. J Clin Invest.

[B12] Carr ME (2001). Diabetes mellitus: a hypercoagulable state. J Diabetes Complications.

[B13] Keren P, George J, Shaish A, Levkovitz H, Janakovic Z, Afek A, Goldberg I, Kopolovic J, Keren G, Harats D (2000). Effect of hyperglycemia and hyperlipidemia on atherosclerosis in LDL receptor-deficient mice: establishment of a combined model and association with heat shock protein 65 immunity. Diabetes.

[B14] Streja D, Cressey P, Rabkin SW (2003). Associations between inflammatory markers, traditional risk factors, and complications in patients with type 2 diabetes mellitus. J Diabetes Complications.

[B15] Guha M, Mackman N (2001). LPS induction of gene expression in human monocytes. Cell Signal.

[B16] Barnes PJ, Karin M (1997). Nuclear factor-kappaB: a pivotal transcription factor in chronic inflammatory diseases. N Engl J Med.

[B17] Arbour NC, Lorenz E, Schutte BC, Zabner J, Kline JN, Jones M, Frees K, Watt JL, Schwartz DA (2000). TLR4 mutations are associated with endotoxin hyporesponsiveness in humans. Nat Genet.

[B18] Kiechl S, Lorenz E, Reindl M, Wiedermann CJ, Oberhollenzer F, Bonora E, Willeit J, Schwartz DA (2002). Toll-like receptor 4 polymorphisms and atherogenesis. N Engl J Med.

[B19] Thorand B, Kolb H, Baumert J, Koenig W, Chambless L, Meisinger C, Illig T, Martin S, Herder C (2005). Elevated levels of interleukin-18 predict the development of type 2 diabetes: results from the MONICA/KORA Augsburg Study, 1984-2002. Diabetes.

[B20] Holle R, Happich M, Lowel H, Wichmann HE (2005). KORA--a research platform for population based health research. Gesundheitswesen.

[B21] National Center for Biotechnology Information SNP database dbSNP. http://www.ncbi.nlm.nih.gov/SNP/.

[B22] Weidinger S, Klopp N, Wagenpfeil S, Rummler L, Schedel M, Kabesch M, Schafer T, Darsow U, Jakob T, Behrendt H, Wichmann HE, Ring J, Illig T (2004). Association of a STAT 6 haplotype with elevated serum IgE levels in a population based cohort of white adults. J Med Genet.

[B23] Meisinger C, Thorand B, Schneider A, Stieber J, Doring A, Lowel H (2002). Sex differences in risk factors for incident type 2 diabetes mellitus: the MONICA Augsburg cohort study. Arch Intern Med.

[B24] Barlow WE (1994). Robust variance estimation for the case-cohort design. Biometrics.

[B25] Thorand B, Baumert J, Kolb H, Meisinger C, Chambless L, Koenig W, Herder C (2007). Sex Differences in the Prediction of Type 2 Diabetes by Inflammatory Markers: Results from the MONICA/KORA Augsburg case-cohort study, 1984-2002. Diabetes Care.

[B26] Schaid DJ, Rowland CM, Tines DE, Jacobson RM, Poland GA (2002). Score tests for association between traits and haplotypes when linkage phase is ambiguous. Am J Hum Genet.

[B27] Li J, Ji L (2005). Adjusting multiple testing in multilocus analyses using the eigenvalues of a correlation matrix. Heredity.

[B28] R Project for Statistical Computing. http://www.R-project.org.

[B29] Illig T, Bongardt F, Schopfer A, Holle R, Muller S, Rathmann W, Koenig W, Meisinger C, Wichmann HE, Kolb H (2003). The endotoxin receptor TLR4 polymorphism is not associated with diabetes or components of the metabolic syndrome. Diabetes.

[B30] Kolek MJ, Carlquist JF, Muhlestein JB, Whiting BM, Horne BD, Bair TL, Anderson JL (2004). Toll-like receptor 4 gene Asp299Gly polymorphism is associated with reductions in vascular inflammation, angiographic coronary artery disease, and clinical diabetes. Am Heart J.

[B31] Rudofsky G, Reismann P, Witte S, Humpert PM, Isermann B, Chavakis T, Tafel J, Nosikov VV, Hamann A, Nawroth P, Bierhaus A (2004). Asp299Gly and Thr399Ile genotypes of the TLR4 gene are associated with a reduced prevalence of diabetic neuropathy in patients with type 2 diabetes. Diabetes Care.

[B32] Mohammad MK, Morran M, Slotterbeck B, Leaman DW, Sun Y, Grafenstein H, Hong SC, McInerney MF (2006). Dysregulated Toll-like receptor expression and signaling in bone marrow-derived macrophages at the onset of diabetes in the non-obese diabetic mouse. Int Immunol.

[B33] Song MJ, Kim KH, Yoon JM, Kim JB (2006). Activation of Toll-like receptor 4 is associated with insulin resistance in adipocytes. Biochem Biophys Res Commun.

[B34] Boekholdt SM, Agema WR, Peters RJ, Zwinderman AH, van der Wall EE, Reitsma PH, Kastelein JJ, Jukema JW (2003). Variants of toll-like receptor 4 modify the efficacy of statin therapy and the risk of cardiovascular events. Circulation.

[B35] Castrillo A, Joseph SB, Vaidya SA, Haberland M, Fogelman AM, Cheng G, Tontonoz P (2003). Crosstalk between LXR and toll-like receptor signaling mediates bacterial and viral antagonism of cholesterol metabolism. Mol Cell.

[B36] Bjorkbacka H, Kunjathoor VV, Moore KJ, Koehn S, Ordija CM, Lee MA, Means T, Halmen K, Luster AD, Golenbock DT, Freeman MW (2004). Reduced atherosclerosis in MyD88-null mice links elevated serum cholesterol levels to activation of innate immunity signaling pathways. Nat Med.

