# Supplementary material for: Association between variations in the TLR4 gene and incident type 2 diabetes is modified by the ratio of total cholesterol to HDL-cholesterol
Source: BMC Med Genet. 2008 Feb 25;9:9. doi: 10.1186/1471-2350-9-9 (PMC2292153; doi:10.1186/1471-2350-9-9)
Supplement: Additional file 1 — Results of interaction effect models including three-way interaction terms between sex, TC/HDL-C and the genetic variants. Results of haplotype and SNP association analysis for men and women combined including three-way interaction terms between sex, TC/HDL-C and the genetic variants. [file 1471-2350-9-9-S1.pdf]

Supplement Table 1 - Results of haplotype analysis in interaction effect models including three-way interaction terms between sex, TC/HDL-C and the genetic variants (n=1,968).

	**β**	**HR**	**[95% CI]**	***p*-value**
H2*	-1.41	0.24	[0.04; 1.51]	0.13
H3*	-1.08	0.34	[0.06; 1.80]	0.20
H4*	-3.74	0.02	[0.00; 0.43]	0.01
H5*	-4.59	0.01	[0.00; 0.22]	3.5×10^{-3}
H6*	0.18	1.20	[0.02; 73.94]	0.93
H7*	-5.80	0.00	[0.00; 0.91]	0.05
Sex*	-2.42	0.09	[0.03; 0.31]	1.6×10^{-4}
TC/HDL-C	-0.48	0.62	[0.45; 0.84]	2.3×10^{-3}
TC/HDL-C× Sex*	0.49	1.63	[1.29; 2.06]	3.7×10^{-5}
Sex × H2*	1.04	2.83	[0.79; 10.12]	0.11
Sex × H3*	1.10	3.01	[0.85; 10.69]	0.09
Sex × H4*	2.42	11.21	[1.73; 72.56]	0.01
Sex × H5*	2.27	9.69	[1.40; 67.11]	0.02
Sex × H6*	-1.03	0.36	[0.01; 9.96]	0.54
Sex × H7*	2.46	11.69	[0.24; 563.09]	0.21
TC/HDL-C × H2*	0.37	1.45	[1.04; 2.01]	0.03
TC/HDL-C × H3*	0.31	1.36	[1.00; 1.84]	0.05
TC/HDL-C × H4*	0.85	2.34	[1.41; 3.88]	1.0×10^{-3}
TC/HDL-C × H5*	0.83	2.30	[1.38; 3.83]	1.3×10^{-3}
TC/HDL-C × H6*	0.03	1.03	[0.52; 2.04]	0.93
TC/HDL-C × H7*	0.93	2.55	[1.18; 5.48]	0.02
Sex × TC/HDL-C × H2*	-0.27	0.76	[0.59; 0.97]	0.03
Sex × TC/HDL-C × H3*	-0.30	0.74	[0.57; 0.97]	0.03
Sex × TC/HDL-C × H4*	-0.59	0.55	[0.39; 0.78]	8.9×10^{-4}
Sex × TC/HDL-C × H5*	-0.40	0.67	[0.47; 0.96]	0.03
Sex × TC/HDL-C × H6*	0.11	1.12	[0.62; 2.03]	0.71
Sex × TC/HDL-C × H7*	-0.40	0.67	[0.37; 1.24]	0.20

* H1 and male sex are used as the reference

Supplement Figure 1 - Hazard ratios of incident type 2 diabetes related to the respective TC/HDL-C concentration in men estimated by interaction effect models including two- and three-way interaction terms between sex, TC/HDL-C and the genetic variants.
Dashed lines represent subjects who are homozygote for the minor allele, while continuous lines show heterozygotes for SNP 1-4 and heterozygotes or homozygotes for the minor allele for SNP 5-7. Small bars at the x-axis indicate TC/HDL-C concentrations of patients with at least one copy of the minor allele.

Supplement Figure 2 - Hazard ratios of incident type 2 diabetes related to the respective TC/HDL-C concentration in men estimated by interaction effect models including two- and three-way interaction terms between sex, TC/HDL-C and the genetic variants.
Dashed lines represent subjects who are homozygote for the minor allele, while continuous lines show heterozygotes for SNP 1-4 and heterozygotes or homozygotes for the minor allele for SNP 5-7. Small bars at the x-axis indicate TC/HDL-C concentrations of patients with at least one copy of the minor allele.

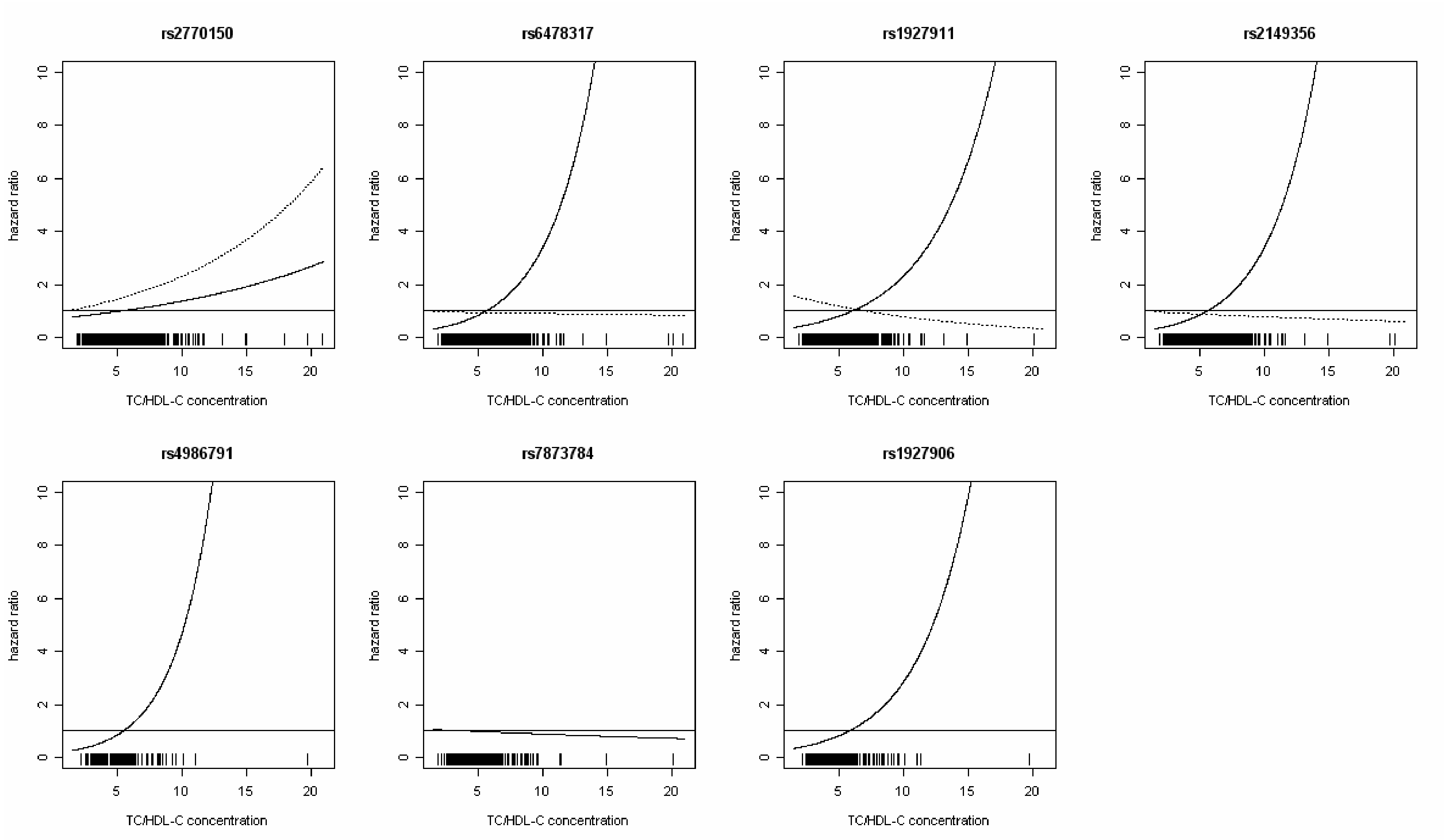

Supplement Figure 1

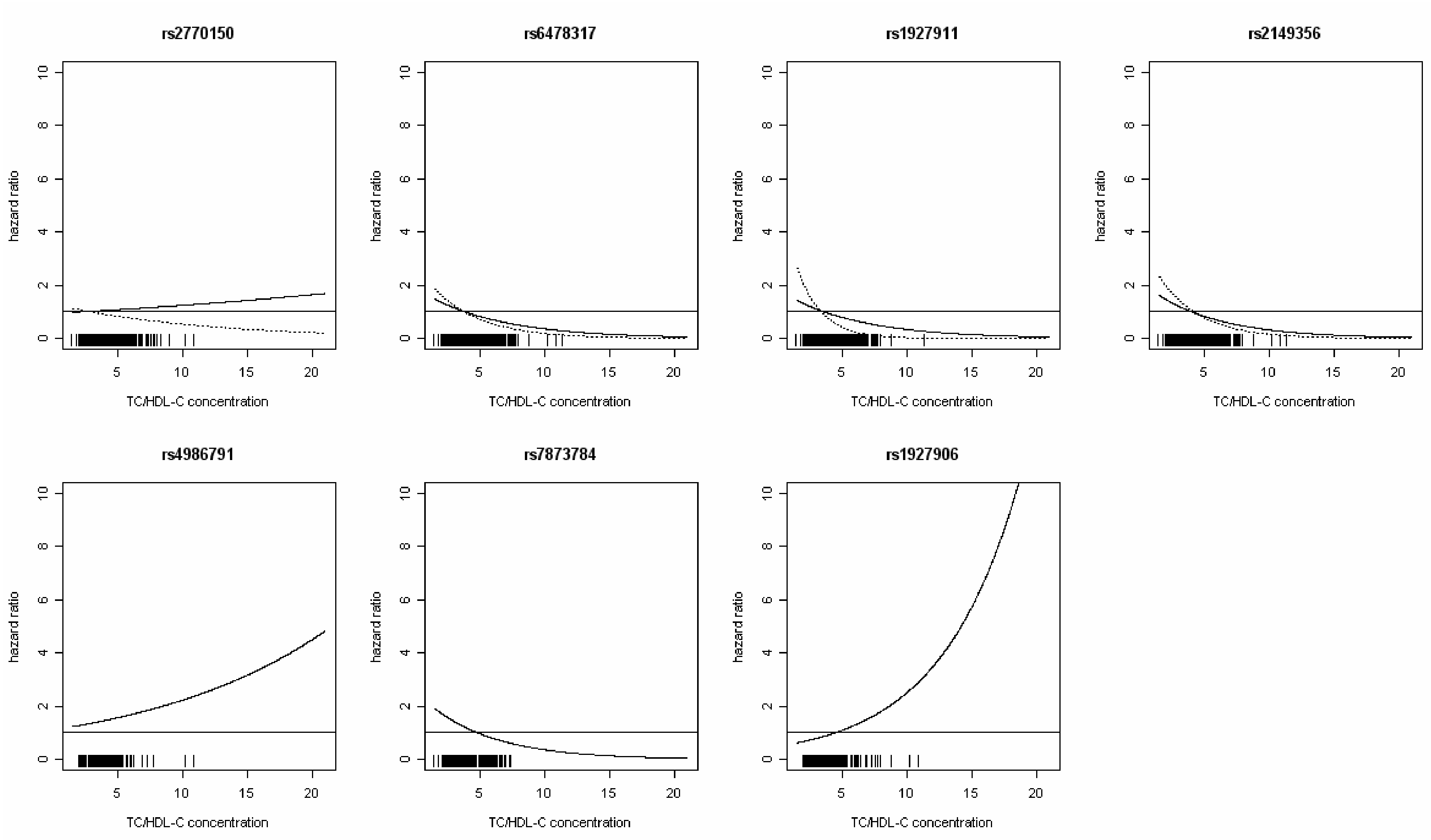

Supplement Figure 2
